# Estrogen receptor alpha inhibits senescence-like phenotype and facilitates transformation induced by oncogenic ras in human mammary epithelial cells

**DOI:** 10.18632/oncotarget.9772

**Published:** 2016-06-01

**Authors:** Zhao Liu, Long Wang, Junhua Yang, Abhik Bandyopadhyay, Virginia Kaklamani, Shui Wang, Lu-Zhe Sun

**Affiliations:** ^1^ Department of Breast Surgery, The First Affiliated Hospital of Nanjing Medical University, Nanjing, China; ^2^ Department of Cellular and Structural Biology, University of Texas Health Science Center at San Antonio, Texas, United States of America; ^3^ Cancer Therapy and Research Center, University of Texas Health Science Center at San Antonio, Texas, United States of America; ^4^ Department of Thyroid and Breast Surgery, Affiliated Hospital of Xuzhou Medical College, Xuzhou, China

**Keywords:** estrogen receptor alpha, senescence, transformation, ras, Gerotarget

## Abstract

Exposure to estrogen has long been associated with an increased risk of developing breast cancer. However, how estrogen signaling promotes breast carcinogenesis remains elusive. Senescence is known as an important protective response to oncogenic events. We aimed to elucidate the role of estrogen receptor alpha (ERα) on senescence in transformed human mammary epithelial cells and breast cancer cells. Our results show that ectopic expression of oncoprotein H-ras-V12 in immortalized human mammary epithelial cells (HMEC) significantly inhibited the phosphorylation of the retinoblastoma protein (Rb) and increased the activity of the senescence-associated beta-galactosidase (SA-β-Gal). These senescence-like phenotypes were reversed by ectopic expression of ERα. Similar inhibition of the H-ras-V12-induced SA-β-Gal activity by ERα was also observed in the human mammary epithelial MCF-10A cells. Co-expression of ERα and H-ras-V12 resulted in HMEC anchorage-independent growth *in vitro* and tumor formation *in vivo*. Furthermore, inhibition of ERα expression induced senescence-like phenotypes in ERα positive human breast cancer cells such as increased activity of SA-β-Gal, decreased phosphorylation of RB, and loss of mitogenic activity. Thus, the suppression of cellular senescence induced by oncogenic signals may be a major mechanism by which ERα promotes breast carcinogenesis.

## INTRODUCTION

Exposure to estrogen is a major risk factor for breast cancer. About two-thirds of breast cancers are estrogen receptor alpha (ERα) positive [1]. However, how estrogen/ERα promotes breast carcinogenesis is not well understood. It is widely believed that estrogen signaling through ERα promotes breast neoplasia by stimulating proliferation of mammary epithelial cells because inhibition of estrogen signaling or deprivation of estrogen can significantly inhibit proliferation of ER positive breast cancer cells. On the other hand, ectopic expression of ERα in ERα negative mammary epithelial cells was shown to inhibit their proliferation [2, 3] and proliferating normal mammary epithelial cells are often ER negative [4]. In fact, high doses of synthetic estrogen were shown to inhibit tumor growth in patients with ER positive breast cancer [5]. These observations suggest that estrogen signaling appears growth-inhibitory in mammary epithelial cells. However, this scenario is inconsistent with the tumorigenesis-promoting activity of estrogen and ER. The controversy surrounding the role of ERα in breast cancer highlights the need for further exploration.

The theory of cellular senescence was proposed by Hayflck and Moorhead in 1961 [6], which is the state of irreversible impairment of cell replication and appears to be influenced intrinsically by telomere shortening. Cellular senescence is one of the mechanisms that normal cells use to avoid carcinogenesis [7]. It was demonstrated that tamoxifen can induce a senescent phenotype in several ERα+ breast cancer cells lines [8], which suggested that ERα might play an important role in the regulation of senescence and growth inhibition in breast cancer.

Herein, we employed a transformed human mammary epithelial cell model to investigate whether ERα expression could inhibit premature cellular senescence. Our results demonstrated that ERα could inhibit oncoprotein-induced premature senescence and consequently facilitates transformation and cell proliferation.

## RESULTS

### Overexpression of ERα and H-ras-V12 in an immortalized human mammary epithelial cell line

**Figure 1:**
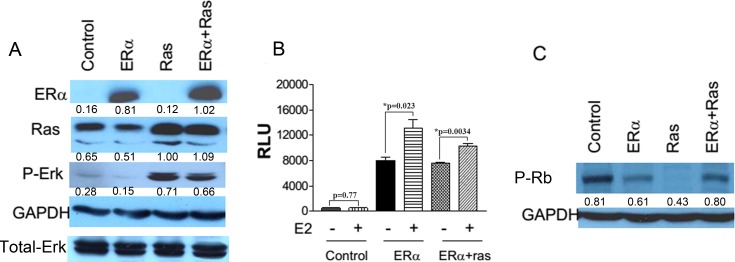
Ectopic expression of ERα and H-ras-V12 in HMEC/hTERT cells hTERT-immortalized HMECs were transduced with an empty retrovirus or a retrovirus carrying an expression cassette of ERα and selected with puromycin. The paired puromycin-resistant cell lines were then transduced with another empty retrovirus (control) or a retrovirus carrying an expression cassette of H-ras-V12. **A.** Protein levels of various transfected and endogenous genes labeled on the left side were detected in the transfected cells listed on the top with Western blot analyses. P-Erk denotes phosphorylated Erk1 and 2. **B.** Control cells and ERα cells without or with H-ras-V12 were co-transfected with an estrogen responsive promoter-luciferase construct and a β-galactosidase expression vector. The cells were then treated without or with 10^−7^M 17β-estradiol for 20h. The cell lysates were used for luciferase and β-gal activity. Relative luciferase activity (RLU) was calculated by normalizing luciferase activity with β-gal activity and plotted. Each value is the mean±SEM from three independent wells. **C.** Phosphorylated Rb protein levels in the transfected cells were detected with Western blot analysis. The value under each band of the Western blots denotes its normalized intensity with the intensity of the corresponding GAPDH or Total-Erk. They were repeated more than once with similar results.

We initially used a human telomerase reverse transcriptase (hTERT)-immortalized human mammary epithelial cell line (HMEC) to determine whether ERα expression can inhibit premature cellular senescence induced by the expression of oncogenic H-ras-V12. HMEC/hTERT cells were first transduced with an empty retrovirus or a retrovirus carrying an expression cassette of ERα and selected with puromycin. The paired puromycin-resistant cell lines were then transduced with another empty retrovirus (control) or a retrovirus carrying an expression cassette of H-ras-V12. The transduced cells were selected with G418. Ectopic expression of these transfected genes were confirmed with Western blot analysis (Figure 1A). Overexpression of H-ras-V12 led to increased phosphorylation of Erk1/2 (Figure 1A) as previously shown [9]. Furthermore, the ectopic ERα was transcriptionally active and responsive to estrogen as demonstrated with increased luciferase activity after transient transfection of a luciferase gene driven by an estrogen responsive promoter into ERα-transfected HMEC/hTERT cells (Figure 1B). These data demonstrate that the transfected proteins were functional.

### Ectopic expression of ERα inhibits premature senescence induced by oncoprotein H-ras-V12

**Figure 2:**
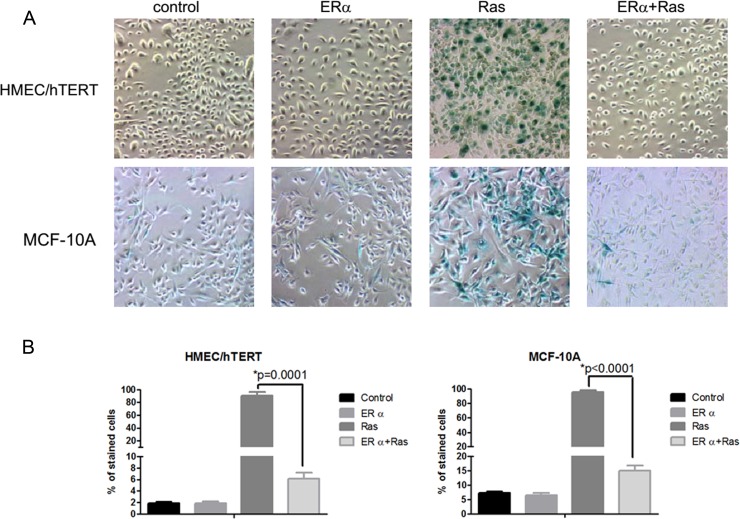
Inhibition of H-ras-V12-induced senescence-like phenotype by ERα **A.** HMEC/hTERT and MCF10A cells were transduced with an empty retrovirus or a retrovirus carrying an expression cassette of ERα. The ERα and control cells were then transduced with a retrovirus carrying an expression cassette of H-ras-V12. The stably transfected cells were stained for SA-β-Gal activity when the H-RAS-V12 cells showed senescence-like morphology. Elevated SA-β-Gal activity was detected in the majority cells transfected with H-ras-V12 as indicated with the dark green/blue staining. **B.**The cells with the dark green/blue staining were counted in four fields and expressed as percent of total number of cells in each field.

Overexpression of H-ras-V12 in control retrovirus-transduced HMEC/hTERT cells led to a drastic ihhibition, often complete arrest, of cell proliferation at early passages after antibiotic selection. In contrast, overexpression of H-ras-V12 in ERα retrovirus-tranduced HMEC/hTERT cells showed no cell cycle arrest. The inhibition of cell division in the H-ras-V12-transfected cells was associated with a marked reduction of phosphorylated Rb protein in comparison with the control and ERα-expressing cells (Figure 1C). The arrested cells appeared larger than ERα-expressing cells and stained positive for senescence-associated beta-galactosidase (SA-β-Gal) suggesting that the expression of H-ras-V12 induced premature senescence (Figure 2A and 2B) as we previously observed [9]. In contrast, the ERα-expressing cells with or without H-ras-V12 stained negative for SA-β-Gal (Figure 2A and 2B). To confirm this observation, we performed the same genetic manipulation in the spontaneously immortalized human mammary epithelial MCF-10A cells. Overexpression of H-ras-V12 gradually induced a senescence-like phenotype during early passages in culture. A population of cells would often emerge from the growth-arrested culture and regain rigorous proliferation as pre-senescent cells. This senescence-like phenotype was repeatedly observed after the expression of H-ras-V12. Similar to the effect of ERα expression in HMEC/hTERT cells, the expression of ERα in MCF-10A cells also prevented H-ras-V12-induced senescence-like phenotype as shown in Figure 2A and 2B.

### Overexpression of H-ras-V12 promotes the tumorigenesis of HMEC

**Figure 3:**
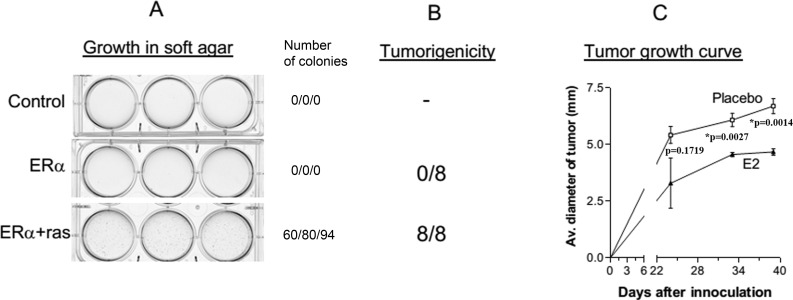
Transformation of HMEC/hTERT cells by ERα and H-ras-V12 **A.** HMEC/hTERT control cells and ERα-transfected cells did not form colonies in soft agarose. In contrast, the cells transfected with ERα and H-ras-V12 were capable of forming colonies in soft agarose. The number of visible colonies were counted in each well and presented in the figure. **B.** The cells transfected with ERα plus H-ras-V12 formed tumors in eight of the eight mice supplemented with or without E2. **C.** Tumor diameter was measured externally in two dimensions using a caliper. The mean diameter of the tumors formed by ERa + H-ras-V12 cells in the mice implanted with the placebo or E2 pellet is plotted as a function of experiment duration. Each data point is the mean+SEM of four tumors in four mice.

The inhibition of H-ras-V12-induced premature senescence by the expression of ERα allowed us to determine whether the cells were transformed. We first determined their ability to grow anchorage-independently in soft agarose, which is an *in vitro* assay for transformation. Because the cells transfected with H-Ras-V12 alone were senescent, they were not used for the assay. As shown in Figure 3A, HMEC/hTERT control cells and ERα-transfected cells did not form colonies in soft agarose. In contrast, the cells transfected with ERα and H-ras-V12 were capable of forming colonies in soft agarose suggesting that the cells were transformed. We next examined their tumorigenicity in ovariectomized female athymic nude mice. HMEC/hTERT cells transfected with ERα alone or ERα plus H-ras-V12 were mixed with Matrigel and BJ fibroblasts, and injected into the fat pad area of the right inguinal mammary gland of the mice. Matrigel and fibroblasts have been shown to promote tumor formation of transformed epithelial cells previously [10–12]. Half of the inoculated mice were implanted with a placebo pellet and the other half were implanted with a 90-day release 17β-estradiol (E2) pellet. Consistent with the soft agarose assay, the ERα-transfected cells were not tumorigenic with or without E2 supplementation (Figure 3B). In contrast, the cells transfected with ERα plus H-ras-V12 formed tumors in all mice regardless of E2 supplementation (Figure 3B). Thus, the cells did not require a high level of estrogen for tumor formation, consistent with the observations that there was a high transactivation activity of ERα in the cells in the absence of estrogen (Figure 1B) and the cells were clonogenic in soft agarose without E2 supplementation (Figure 3A). Interestingly, supplementation with E2 significantly inhibited tumor growth in nude mice (Figure 3C).

### Knockdown of ERα expression induces senescence-like phenotypes in ERα positive breast cancer cells

**Figure 4:**
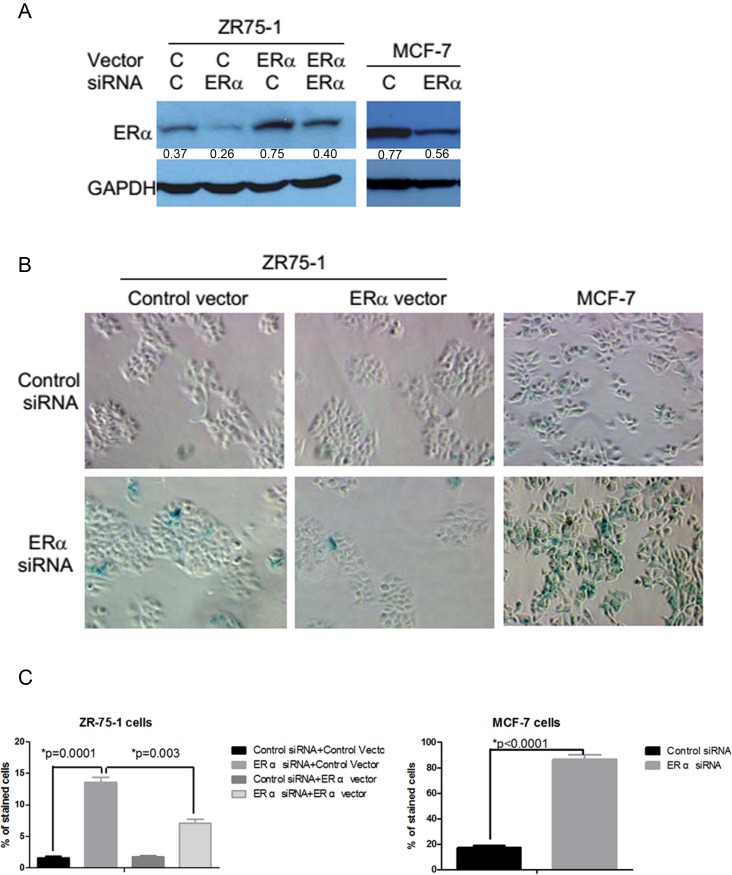
Induction of senescence-like phenotypes by the inhibition of ERα expression in ERα positive breast cancer cells **A.** ZR75-1 and MCF-7 cells were transiently tranfected with a control siRNA or a mixture of four ERα siRNAs at 50 nM. To determine whether the senescence-like phenotypes induced by ERα siRNA is specifically due to the inhibition of ERα, ZR75-1 cells were also transfected with a control or an ERα cDNA expression plasmid at 0.43 nM with or without ERα siRNAs. The transfected cells were lysed after 6 days and cell extracts were used in Western blots. The value under each band denotes its normalized intensity with the intensity of the corresponding GAPDH. The experiments were repeated two times with similar results. **B.** SA-β-gal staining was performed after transfection in ZR75-1 cells and MCF-7 cells. **C.** The cells with the dark green/blue staining were counted in four fields and expressed as percent of total number of cells in each field.

**Figure 5:**
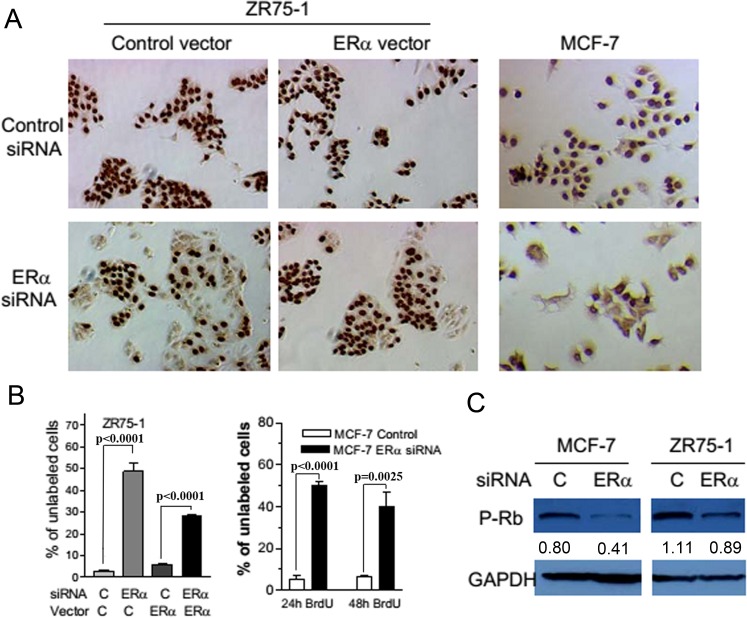
The suppression of ERα expression inhibited DNA synthesis and reduced the phosphorylation of Rb in breast cancer cells **A.** BrdU was added to ZR75-1 cells and MCF-7 cells. An anti-BrdU antibody was used to detect BrdU incorporated in the nuclear DNA. **B.** Images of four random high power fields at 100X magnifications were captured from each group in panel A. The cells that are not labeled with BrdU were counted and expressed as percent of total number of cells in each field. Each column is the mean±SEM from the four high power fields. For MCF-7 cells, BrdU was incubated with the transfected cells for 24 as well as 48 hours to ascertain that the arrest of cell cycle progression was not a transient phenomenon. **C.** Cells transfected with the control or ERa siRNA were lysed after two days and cell extracts with 40 mg total protein were used in the West blot analysis of phosphorylated Rb. GAPDH was used to indicate equal loading. The value under each band denotes its normalized intensity with the intensity of the corresponding GAPDH. The experiments were repeated two times with similar results.

To determine whether the endogenous ERα played a similar role in spontaneously transformed mammary epithelial cells, we used an RNA interference approach to knock down the expression of ERα in ERα positive human breast cancer cells. Transient transfection of a mixture of ERα small interfering RNAs (siRNAs) containing four different sequences of siRNA was shown to significantly reduce ERα protein levels in the ZR75-1 and MCF-7 breast cancer cells and the ERα level could be restored by co-transfection with an ERα expression plasmid (Figure 4A). The inhibition of ERα expression with the transient transfection of the siRNAs induced senescence-like phenotypes in ERα positive breast cancer cells. Many ERα siRNA-transfected ZR75-1 and MCF-7 cells showed strongly positive SA-β-Gal activity (Figure 4B). The induction of SA-β-Gal was specifically due to the inhibition of ERα expression because co-transfection of ERα siRNAs with an ERα expression plasmid inhibited SA-β-Gal expression in ZR75-1 cells (Figure 4B and 4C). Similar results were obtained in another ERα positive breast cancer T47D cells (data not shown). The inhibition of ERα expression also inhibited DNA synthesis as reflected by the lack of BrdU incorporation in about half of ZR75-1 and MCF-7 cells transiently transfected with ERα siRNAs (Figure 5A and 5B). Again, ectopic expression of ERα was able to attenuate the inhibitory effect of ERα siRNA in DNA synthesis (Figure 5A and 5B). These observations are consistent with data demonstrating the inhibition of cell cycle by antiestrogens [13]. However, it should be noted that the cessation of DNA synthesis was observed in the cells that were cultured in a fully growth-promoting medium after several days of ERα siRNA transfection. Furthermore, the percentage of unlabeled cells did not significantly change even after 48 hr incubation with BrdU in comparison with that after 24 hr incubation with BrdU in ERα siRNA transfected MCF-7 cells (Figure 5B) and ZR75-1 cells (data not shown). The reduced BrdU incorporation was associated with reduced phosphorylated Rb levels in both MCF-7 and ZR75-1 cells with ERα knockdown (Figure 5C). These features are consistent with the senescent phenotype.

## DISCUSSION

Long term exposure to estrogen is associated with an increased risk of breast cancer. Estradiol administration induced breast cancer formation in various animal models and anti-estrogens abrogated this effect [14, 15]. Although ERα has been extensively studied in breast cancer, how estrogen signaling promotes breast carcinogenesis remains elusive. In general, it is widely accepted that ERα binding to the promoters of its target genes can lead to increased expression of growth promoting genes and decreased expression of growth inhibitory genes [16]. Yet, ERα is considered to be a good prognostic indicator for breast cancer survival and loss of ERα is associated with cancer progression and poor prognosis [17] [18]. Moreover, our previous study indicated that stable reintroduction of functional ERα in ERα-negative human breast cancer cells can inhibit their aggressive bone-metastatic potential [19]. Therefore the mechanism by which ERα promotes breast carcinogenesis is unsettled.

The accumulation of oncogenic signals due to genetic mutations and epigenetic alterations is known to drive the tumorigenesis of breast epithelial cells. However, oncogenic signals from oncogenes such as the constitutively active H-ras-V12 can lead to a permanent cell cycle arrest, also known as senescence-like growth arrest or premature cellular senescence. [9, 20]. This type of senescence suppresses cellular transformation and acts as a potent protective response to oncogenic events during early stage of neoplastic transformation by casting out the transformed cells [7]. Consistent with these previous reports, our data showed that overexpression of H-ras-V12 in human mammary epithelial cells (HMEC) and the spontaneously immortalized human mammary epithelial cells MCF-10A caused remarkable senescence-like growth arrest.

It was reported that senescence in ERα-dependent breast cancer cells induced by tamoxifen appeared to result from the downregulation of survival signals generated by the transcriptional activity of ERα, indicating an anti-senescence role of ERα in breast cancer [8]. Consistently, our results demonstrated that the knockdown of ERα expression with the transfection of the siRNAs induced senescence-like phenotypes in ZR-75-1 and MCF-7 cells. Furthermore, our observations suggested that the exit from cell cycle due to the down-regulation of ERα was long-lasting and resembled premature senescence. Consistent with the phenotype of premature senescence, we also observed a reduction of phosphorylated Rb protein in ERα siRNA transfected MCF-7 and ZR75-1 cells as has been shown in other cellular senescence models [21]. Moreover, the ectopic expression of ERα was shown to restore the high level of P-Rb in HMECs, which indicates that ERα appears to regulate senescence through modulation of Rb phosphorylation in breast epithelial cells. Further studies are needed to validate our preclinical findings with clinical specimens from a cohort of surgical tissue samples from patients with ERα positive breast cancer, who underwent neoadjuvant endocrine therapy before surgery, to investigate whether ER directed therapy induces senescence *in vivo*.

In the present study, we demonstrated that the overexpression of ERα attenuated the premature senescence induced by H-ras-V12 in both HMEC and MCF-10A cells. Ras is a small GTPase protein, which plays critical regulatory roles in the proliferation, differentiation, survival, and transformation of normal cells [22]. To confirm whether the HMECs transfected with H-ras-V12 were transformed, we performed the soft agarose assay. Notably, we found that the HMEC/hTERT cells transfected with ERα and H-ras-V12 were capable of forming colonies in soft agarose. Moreover, the cells transfected with ERα and H-ras-V12 formed tumors in ovariectomized female athymic nude mice suggesting that the cells were transformed. We have shown that the senescence-like phenotype inhibited by ER happened in both earlier stages of breast cancer (transformed HMEC) and later stages (ZR-75-1 and MCF-7). However, the effects in metastatic breast cancer has not been verified. Further studies are needed to investigate the role of senescence regulated by ERα in metastatic breast cancer.

Because of the anti-senescence role of ERα in both H-ras-V12-transformed breast epithelial cells and breast cancer cells described above, we assumed that estrogen might be able to stimulate the tumor growth by HMEC transformed with ERα and H-ras-V12. Interestingly, the supplementation with 17β-estradiol significantly inhibited tumor growth in nude mice. This observation appears consistent with several published observations. Estrogen treatment inhibited the growth of ERα negative breast epithelial cells after ERα was ectopically expressed in these cells [2, 3]. We and others have also shown that ectopic expression of ERα inhibited ERα negative breast cancer cell invasion and metastasis [19, 23]. It was reported that high doses of estrogen could inhibit the progression of breast cancer in some patients with exhaustive antiestrogen therapy. [5]. Further studies confirmed that estrogen induced growth suppression in endocrine resistant breast cancer was due to apoptosis [24, 25]. However, the mechanisms for the growth inhibition by estrogen in the endocrine resistant breast cancer are likely different from the growth inhibition by estrogen in the ERα-transfected cells we observed. In addition, estrogen could also activate ERβ, which is known to mediate a growth inhibitory response *in vitro* and *in vivo*. Thus, the data from published studies and our current study appear to suggest that while ERα expression can mediate the escape of premature senescence induced by oncogenic Ras, further activation of estrogen receptor(s) by estrogen treatment can inhibit the growth of ERα negative breast epithelial cells after ectopic expression of ERα. Further studies are clearly needed to generalize this concept.

In summary, our study showed that ERα can inhibit premature senescence and consequently facilitate transformation induced by oncogenic Ras protein, and that knocking down ERα induced senescence-like phenotypes in ERα positive breast cancer cells. Our observations also suggested that the stimulation of breast cancer cell proliferation by estrogen and ERα might be in part due to the inhibition of senescence-like growth arrest induced by cellular oncogenic signals that cause malignant transformation of ERα positive breast cancer cells.

## MATERIALS AND METHODS

### Ethics statement

All animal protocols were approved by the Institutional Animal Care and Use Committee of the University of Texas Health Science Center at San Antonio. All animal experiments were monitored by the Department of Laboratory Animal Resources at the University of Texas Health Science Center at San Antonio

### Cell lines and transfection

Human mammary epithelial cells (HMECs) from Cambrex were cultured in MEGM from Cambrex. The spontaneously immortalized human mammary epithelial MCF-10A cells were obtained from the Michigan Cancer Foundation and cultured in the DMEM/F-12 medium originally used to develop the cell line [26]. Human breast cancer ZR75-1 and MCF-7 cell lines were obtained from ATCC and the Michigan Cancer Foundation, respectively, and cultured in a supplemental McCoy's 5A medium containing 10% fetal bovine serum [27]. Retroviral packaging PA317 cells were obtained from ATCC and cultured in DMEM containing 10% fetal bovine serum. Human foreskin fibroblast BJ cells were obtained from Dr. Olivia Pereira-Smith and cultured in MEM containing 10% fetal bovine serum. Working cultures were maintained at 37°C in a humidified incubator with 5% CO_2_.

Stable ectopic expression of various genes was accomplished with retrovirus infection. Retroviral expression vectors of hTERT, ERα, and H-ras-V12 were individually transfected into PA317 cells with FuGENE 6 (Roche). Each vector carried an antibiotic resistance gene. Conditioned media containing the replication-incompetent retroviruses were added to exponentially growing target cell cultures. After two days of incubation, the infected cells were selected with one of the three antibiotics, G418 at 400 μg/ml, puromycin at 1 μg/ml, or hygromycin at 10 μg/ml for HMECs or 200 μg/ml for MCF-10A cells. HMECs were immortalized with ectopic expression of hTERT as previously described [10]. Two HMEC/hTERT cell lines, the parental HMEC/hTERT cell line and one of its limiting dilution clones, were used in experiments. Both cell lines yielded identical results with respect to the inhibition of H-ras-V12-induced senescence by ectopic expression of ERα. Therefore, only one set of data from either of the two cell lines is presented.

To determine the effect of ERα on H-ras-V12-induced premature senescence, HMEC/hTERT and MCF-10A cells were first transduced with an empty retrovirus or a retrovirus carrying an expression cassette of ERα and selected with puromycin. The paired puromycin-resistant cell lines were then transduced with another empty retrovirus (control) or a retrovirus carrying an expression cassette of H-ras-V12. The transduced cells were selected with G418.

For transient transfection of an ERα expression plasmid and/or ERα siRNAs (Dharmacon), ZR75-1 and MCF-7 cells were plated at 0.3-0.7×10^6^ cells per well of a 6-well plate for Western blot analyses or 0.6-1.2×10^5^ cells per well of a 24-well plate for senescence-associated beta-galactosidase assays and BrdU incorporation assays. The cells were transfected with 50 nM control or ERα siRNA mixture containing 4 different sequences of siRNA in the presence or absence of 0.43 nM control or ERα expression plasmid mixed with Lipofectamine 2000 (Invitrogen). The transfected cells were used for various analyses after 2 to 6 days.

### Western blot

Cells were rinsed two times with ice-cold PBS and lysed in a lysis buffer (50 mM Tris-HCl at pH 7.4, 150 mM NaCl, and 1% Nonidet P-40) containing protease inhibitors (Roche) and phosphatase inhibitors (1 mM NaVO_3_ and 1mM NaF). Forty to seventy micrograms of cell lysate protein were then separated with SDS-PAGE and transferred to a PVDF membrane (Amersham Biosciences). The membrane was blocked with TBST (100 mM Tris-HCl at pH 8.0, 150 mM NaCl, 0.05% Tween-20) containing 5% non-fat dried milk and then incubated with various primary antibodies for 1 hour. After three washes with TBST, the membrane was incubated with a HRP-linked anti-rabbit or anti-mouse secondary antibody (Santa Cruz) for 1 hour and washed again. Bound complexes were visualized using chemiluminescence procedures (NEN Life Science Products) and autoradiography. The primary antibodies used include anti-ERα mAb (Lab Vision), anti-ras-V12 mAb (Oncogene), anti-p53 mAb (Santa Cruz), anti-phospho-Erk1/2 polyclonal Ab (Cell Signaling), anti-phospho-Rb polyclonal Ab and anti- total-Rb polyclonal Ab (Cell Signaling), and anti-GAPDH mAb (Ambion). The density of representative images was quantified using Photoshop software. Phosphorylated proteins was normalized by their total proteins or by GAPDH.

### Estrogen responsive promoter assay

To determine the transcriptional activity of the ectopically transfected ERα, a TK-ERE-luciferase construct (0.5 μg) was co-transfected with a β-galactosidase construct (0.1 μg) into the control and ERα-transfected HMECs plated in a 12-well plate with FuGENE 6. The transfected cells were treated with or without 10^−7^ M 17β-estradiol for 20 h. The cell lysates were used for luciferase and β-galactosidase assays as previously described [28]. Relative luciferase activity (RLU) was calculated by normalizing luciferase activity with β-galactosidase activity.

### Senescence-associated beta-galactosidase (SA-β-Gal) assay

SA-β-Gal assay was performed following the procedure described by Dimri et al [29]. Briefly, sub-confluent cultures of various cells were fixed with 3% formaldehyde for 5 min and then incubated with a staining solution containing 0.5 mg x-gal per milliliter of PBS for 3 hours to overnight. Senescent cells containing SA-β-Gal activity were stained with a blue color.

### Soft agarose assay

To assess the *in vitro* tumorigenic potential of the cells transfected with ERα or ERα plus H-ras-V12, their anchorage-independent growth ability in soft agarose was examined as described previously [28]. Briefly, exponentially growing cells were suspended at 6,000 cells/ml of 0.4% low melting point agarose dissolved in the culture medium and plated on the top of a 1-ml underlayer of 0.8% agarose in each well of a 6-well plate. After 3 weeks of incubation at 37°C in a humidified incubator with 5% CO_2_, cell colonies were visualized by staining with 1 ml of *p*-iodonitrotetrozolium violet.

### *in vivo* tumorigenicity study

To determine whether ectopic expression of ERα and H-ras-V12 can induce the HMEC/hTERT cells to form tumors *in vivo*, exponentially growing HMEC/hTERT cells transfected with ERα alone or with H-ras-V12 were mixed with BJ fibroblasts and Matrigel (Collaborative Research) and injected into the fat pad area of the right inguinal mammary gland of ovariectomized 5-wk-old female nude mice (Harlan Sprague Dawley, Inc.). Each injection contained 6.7 million HMEC, 1.6 million BJ cells and 50 μl Matrigel in a total of 100 μl volume. Half of the inoculated mice (4 for each group) were implanted with a placebo pellet and the other half were implanted with a 90-day release 17β-estradiol (E2) pellet (Innovative Research of America). Tumor size was measured externally in two dimensions with a caliper. Average tumor diameter was calculated by dividing the sum of tumor length and width by two. At the termination of the experiment, animals were euthanized with carbon dioxide inhalation followed by cervical dislocation.

### DNA synthesis assay

Mitogenic activity as a function of ERα expression level was determined by measuring BrdU incorporation in nuclear DNA. ERα positive human breast cancer cells were plated in a 24-well plate and transfected with ERα siRNA and/or the ERα expression plasmid. After two to five days, BrdU was added to the transfected cells at a final concentration of 10 μM. After an additional 24 or 48 hr incubation, the cells were washed twice with PBS and fixed in 70% ethanol for 20 min at 4°C. The fixed cells were treated with 2.5 N HCl for 20 min, with 3% H_2_O_2_ for 10 min, and with 100 mM boric acid for 6 min. BrdU incorporated in nuclear DNA was stained in brown color by incubating the treated cells with an anti-BrdU antibody (BD PharMingen) and with the Vectastain ABC kit and DAB kit from Vector Laboratories, Inc. according to the manufacturer's instruction.

### Statistical analysis

Two-tailed student t-tests or one way ANOVA were performed to determine the significant difference between control and experimental data. All the statistical analyses were performed using STATA11(StataCorp LP, College Station, TX, USA.) A p value of less than 0.05 was considered statistically significant.

## Abbreviations

ERα: estrogen receptor alpha
HMEC: human mammary epithelial cells
RLU: Relative luciferase activity
hTERT: human telomerase reverse transcriptase
SA-β-Gal: senescence-associated beta-galactosidase, E2=17β-estradiol
